# Supplementary material for: A flexible ultrasensitive optoelectronic sensor array for neuromorphic vision systems
Source: Nat Commun. 2021 Mar 19;12:1798. doi: 10.1038/s41467-021-22047-w (PMC7979753; doi:10.1038/s41467-021-22047-w)
Supplement: Supplementary file 1 — Supplementary Information [file 41467_2021_22047_MOESM1_ESM.pdf]

Supplementary Materials for

A Flexible Ultrasensitive Optoelectronic Sensor Array for Neuromorphic Vision Systems

Qian-Bing Zhu[1,2,9], Bo Li[1,2,9], Dan-Dan Yang[3], Chi Liu[1], Shun Feng [1,4], Mao-Lin Chen[1], Yun Sun[1], Ya-Nan Tian[5], Xin Su[6], Xiao-Mu Wang[6], Song Qiu[7]*, Qing-Wen Li[7], Xiao-Ming Li[3]*, Hai-Bo Zeng[3], Hui-Ming Cheng[1,2,8]*, Dong-Ming Sun[1,2]*

[1]Shenyang National Laboratory for Materials Science, Institute of Metal Research, Chinese Academy of Sciences, 72 Wenhua Road, Shenyang, 110016, China.

[2]School of Material Science and Engineering, University of Science and Technology of China, 96 Jinzhai Road, Hefei, 230026, China.

[3]College of Materials Science and Engineering, Nanjing University of Science and Technology, 200 Xiaolingwei Street, Nanjing, 210094, China.

[4]School of Physical Science and Technology, ShanghaiTech University, 393 Huaxiazhong Road, Shanghai, 200031, China.

[5]College of Information Science and Engineering, Northeastern University, 3-11 Wenhua Road, Shenyang, 110819, China.

[6] School of Electronic Science and Engineering, Nanjing University, Nanjing, 210093, China.

[7]Suzhou Institute of Nano-Tech and Nano-Bionics, Chinese Academy of Sciences, 398 Ruoshui Road, Suzhou, 215123, China.

[8]Tsinghua-Berkeley Shenzhen Institute, Tsinghua University, 1001 Xueyuan Road, Shenzhen, 518055, China.

[9]These authors contribute equally：Qian-Bing Zhu, Bo Li.

Email: sqiu2010@sinano.ac.cn; lixiaoming@njust.edu.cn; cheng@imr.ac.cn; dmsun@imr.ac.cn

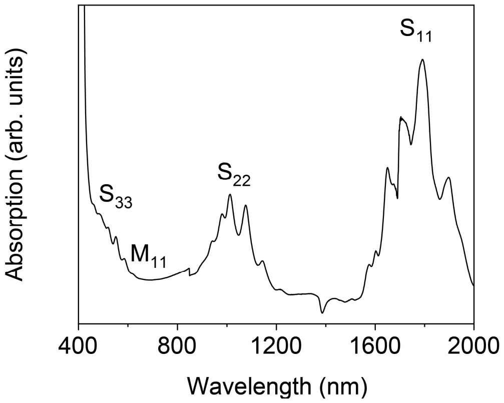

Supplementary Fig. 1 UV–Vis–NIR spectrum of the purified CNTs. According to the UV – Vis – NIR spectrum of the supernatants, only the S_{11} and S_{22} peaks (semiconducting CNTs) are observed without metallic CNTs (M_{11} peaks), indicating the ultra-high content of semiconducting CNTs.

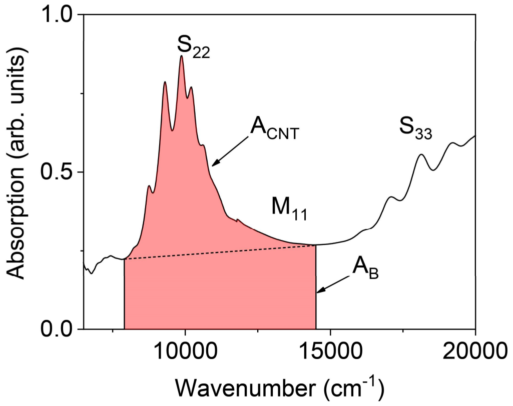

Supplementary Fig. 2 Absorption spectrum of the purified semiconducting-CNTs for calculating absorption peak ratio (ϕ_i). The purity of semiconducting-CNTs is evaluated from the absorption spectrum on a reported method[1]. This method is applicable for the removal of the M_{11} peak due to the elimination of metallic CNTs by calculated absorption peak ratio (ϕ_i). $\phi_i = A_{\mathrm{CNT}}/(A_{\mathrm{CNT}} + A_{\mathrm{B}}) = 0.420$, where A_{CNT} (red area) is the enveloping area of the M_{11} and S_{22} bands enclosed by the linear baseline (dot line) corresponding to the amount of metallic- and semiconducting-CNTs in the sample, and A_{B} is the area covered by the linear baseline of the same region, mainly attributing to the amorphous carbon impurity. The purity of semiconducting-CNTs in our work exceeds 99.9%, because ϕ_i in our work is larger than that (0.404) in previous work reported by J. Ding et al. It should be noted that the spectral-based evaluation methods are not accurate enough to characterize the semiconducting-CNTs with a purity higher than 99%. Through electrical measurements of short-channel transistors, the purity of semiconducting-CNTs sorted using the same homopolymer (PCz) can reach more than 99.9%[2, 3]. In our study, the transfer characteristics of 1024 transistors in the sensor array also proved the high purity of semiconducting-CNTs (Supplementary Fig. 29)

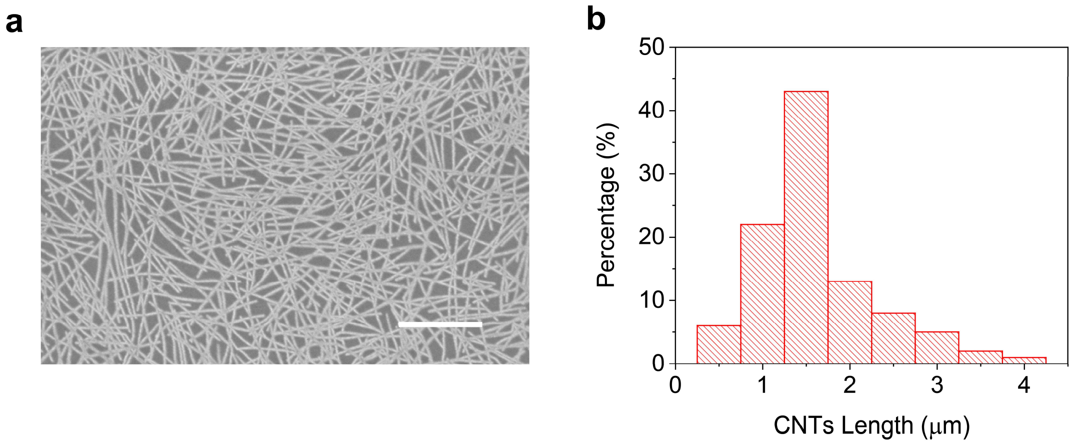

Supplementary Fig. 3 Morphology and CNT length distribution of semiconducting-CNT film. (a) SEM image of semiconducting-CNT film with a deposition time of 30 minutes (scale bar, 1 μm). (b) Histogram of statistical distribution of the length of semiconducting-CNTs with a mean length of CNTs is 1.4 μm.

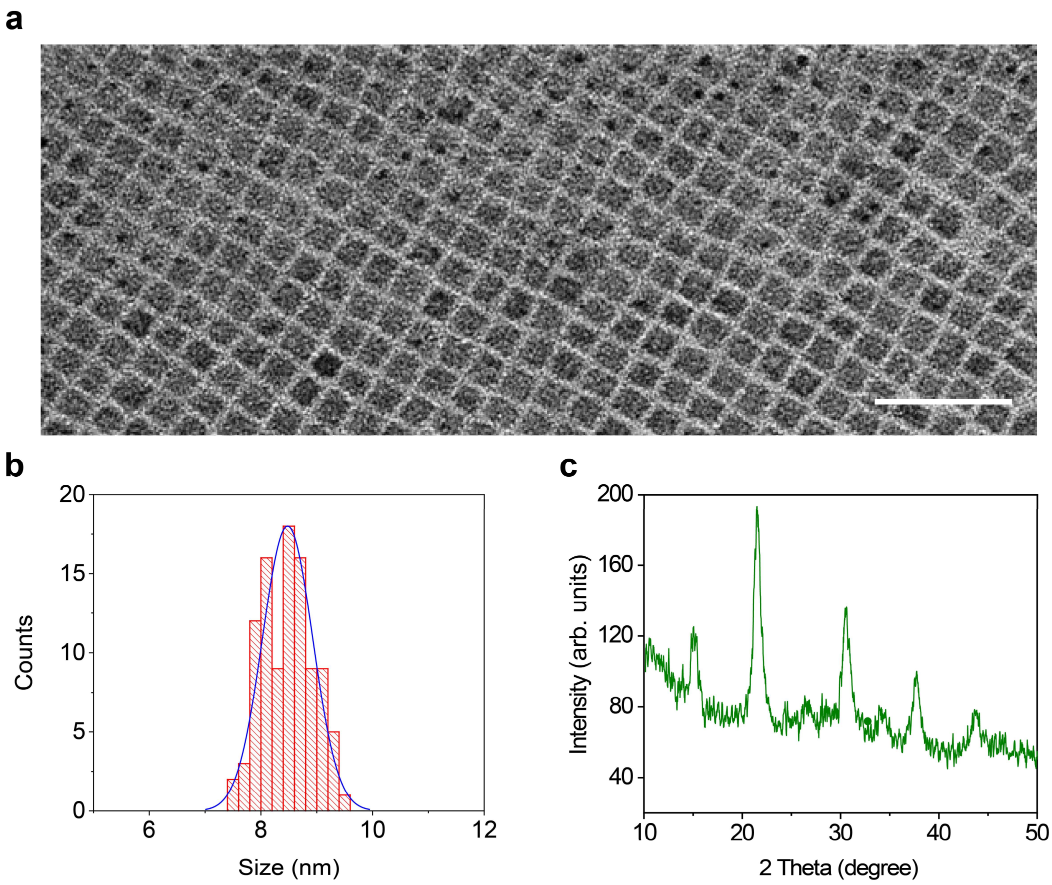

Supplementary Fig. 4 Characterization of $CsPbBr_3$-QDs. (a) TEM image of $CsPbBr_3$-QDs (scale bar, 40 nm). (b) Histogram of statistical distribution of the size of $CsPbBr_3$-QDs. The $CsPbBr_3$-QDs exhibit a cubic crystal structure with a mean size of ~8.5 nm. (c) XRD characteristics.

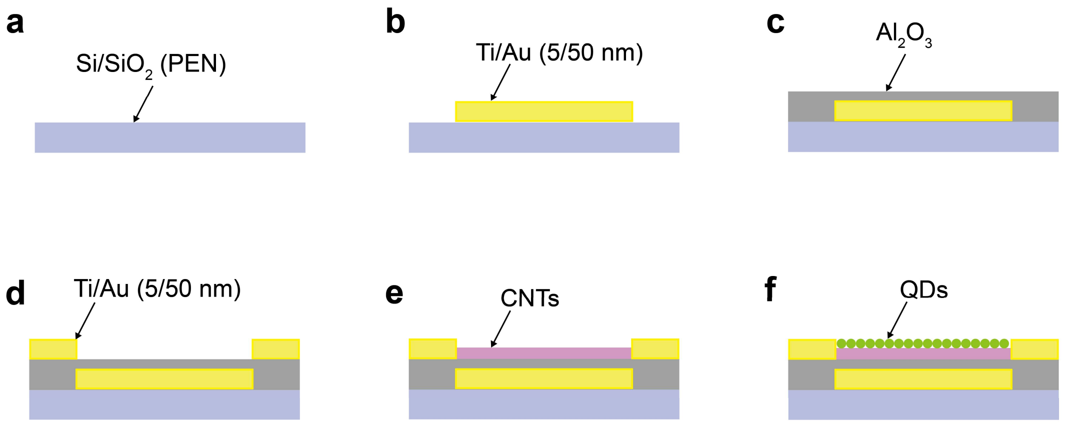

Supplementary Fig. 5 Device fabrication process. (a) An initial SiO_2/Si or PEN substrate. (b) Gate electrode fabrication. The gate electrode (Ti/Au: 5/50 nm) was formatted by standard photolithography, e-beam evaporation and lift-off. (c) Insulator layer fabrication. A 30-nm-thick Al_2O_3 layer was fabricated on the substrate by an atomic layer deposition (ALD) technique (trimethylaluminum and water as precursors, 150 °C). (d) Source/drain electrode fabrication. The source and drain electrodes were formatted on the insulator by the aforementioned method. (e) Deposition and patterning of a solution-processed CNT channel. (f) Deposition of a $CsPbBr_3$-QD layer. The $CsPbBr_3$-QDs were spin coated on the top surface of the channel.

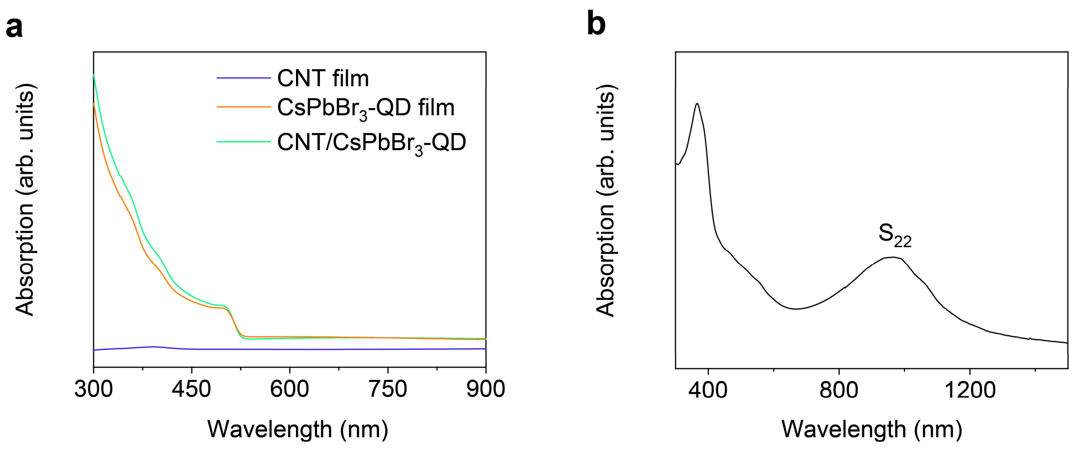

Supplementary Fig. 6 Absorption spectrum. (a) Absorption spectra of the CNT film, $CsPbBr_3$-QD film and CNT/$CsPbBr_3$-QD film on a quartz substrate. Because the thickness of the CNT channel is only 2-5 nm, the absorption of CNT film in the region of 300-900 nm is relatively weak. The all-inorganic perovskite $CsPbBr_3$-QD film can easily cover the absorption of the CNTs due to its excellent light absorption properties. Note that there is no obvious change between the hybrid film and the $CsPbBr_3$-QD film. (b) Absorption spectrum of a thick CNT film (50 nm).

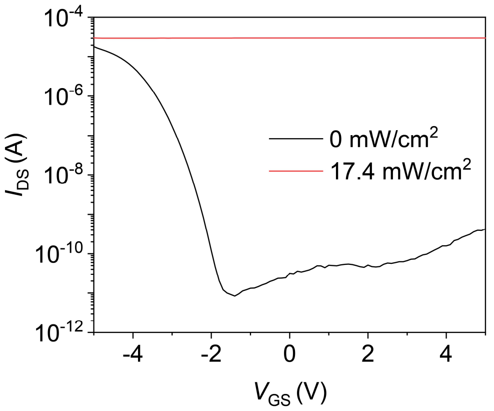

Supplementary Fig. 7 I_{DS}-V_{GS} curves of the CNT/$CsPbBr_3$-QD phototransistor under dark and illuminated conditions (P = 17.4 mW/cm^2). The accurate value of the on- and off-current ratio is evaluated to be 3.6×10^6. V_{DS} = 1 V, λ = 506 nm.

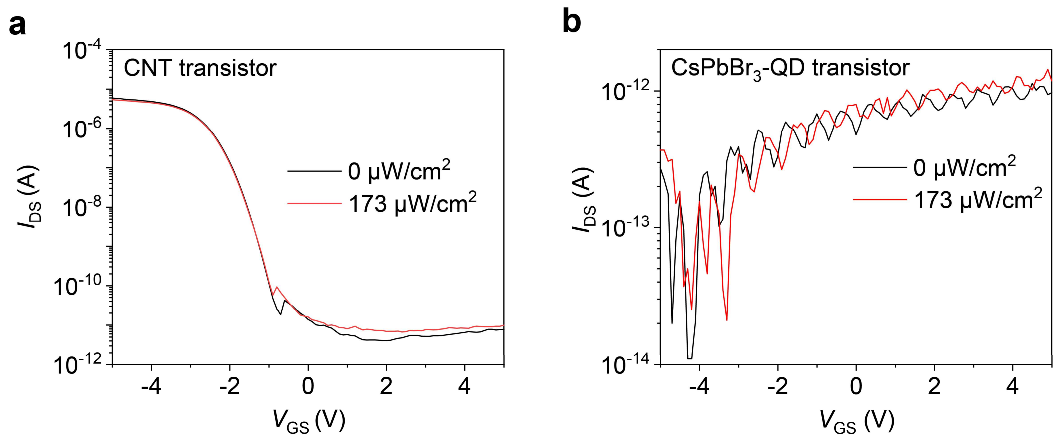

Supplementary Fig. 8 Electrical and optoelectronic performances of CNT transistor and $CsPbBr_3$-QD transistor. (a) I_{DS}-V_{GS} characteristics of CNT transistor under dark and illuminated conditions (P = 173 μW/cm^2). The I_{DS}-V_{GS} characteristics of CNT transistor show normal electrical performance with an on- and off-current ratio of 10^6, but there is almost no optical response for a laser with a wavelength of 405 nm and a power density of 173 μW/cm^2 (b) I_{DS}-V_{GS} characteristics of $CsPbBr_3$-QD transistor under dark and illuminated conditions (P = 173 μW/cm^2). The inherent carrier mobility of $CsPbBr_3$-QDs is very low, and there is a large contact resistance between adjacent $CsPbBr_3$-QDs, thus the $CsPbBr_3$-QD film exhibits non-conductive behavior. V_{DS} = 1 V, λ = 405 nm.

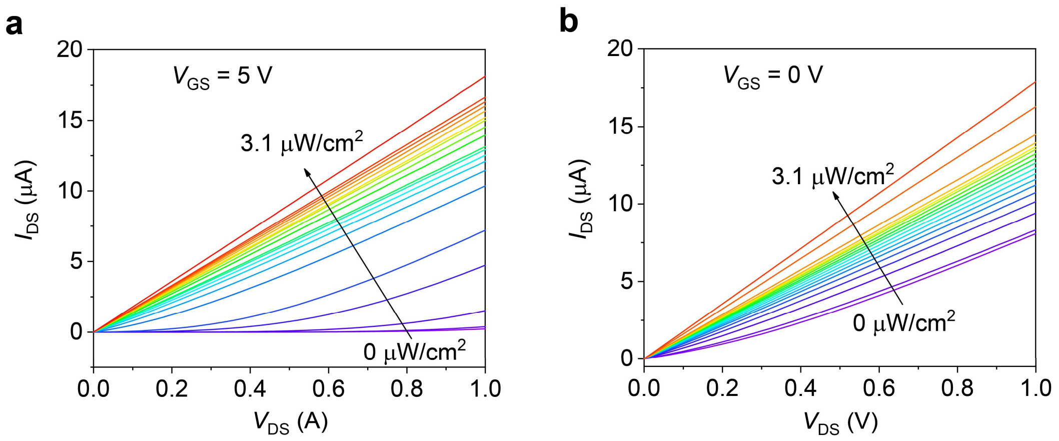

Supplementary Fig. 9 I_{DS}-V_{DS} curves under various lighting power densities with a fixed V_{GS}. (a) V_{GS} = 5 V. (b) V_{GS} = 0 V. λ = 405 nm.

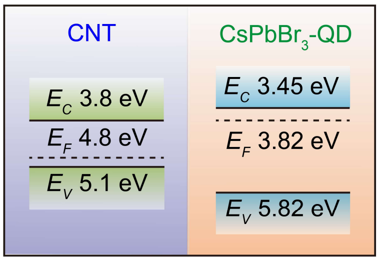

Supplementary Fig. 10 Band structure of CNT and $CsPbBr_3$-QD. According to the absorption spectrum of the purified CNTs (Supplementary Fig. 1), the S_{22} peak can be determined to be ~1000 nm, which can be inferred from the empirical Kataura plot[4] that the band gap of the semiconducting-CNT is ~1.3 eV. Meanwhile, we can conclude that the valance band is about 5.1 eV[5] and the conduction band can be inferred as 3.8 eV. And the energy band diagram includes more information including the work function of CNT of ~4.8 eV[6] and the band structure of $CsPbBr_3$-QD[7].

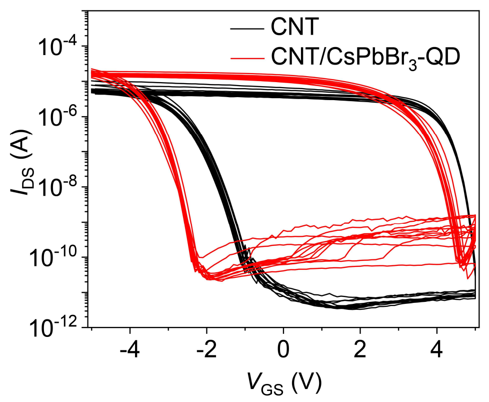

Supplementary Fig. 11 Transfer characteristics of 12 transistors with the CNT channels and CNT/$CsPbBr_3$-QD in ambient dark conditions. When $CsPbBr_3$-QDs are spin-coated on the CNT channel, the carriers (electrons) flow from $CsPbBr_3$-QDs to CNTs due to the difference of the Fermi levels, thereby leading to a built-in electric field in the space charge region, where the $CsPbBr_3$-QD side has a higher voltage potential (Supplementary Fig. 10 and Fig. 1e). This built-in electric field is the key to effectively splitting photo-generated carriers[8-10]. Due to the opposite electric force applied on them, the photo-generated electrons and holes formed in the space charge region will be separated by the built-in electric field. The holes flow to the CNT side with a lower voltage potential while the electrons flow to the $CsPbBr_3$-QD side with a higher voltage potential and are trapped (Fig. 1e). This process can also be verified by the negative shift of the I_{DS}-V_{GS} characteristics and the transient PL spectra of $CsPbBr_3$-QD and CNT/$CsPbBr_3$-QD films (Supplementary Fig. 12b). When $CsPbBr_3$-QDs are spin-coated, the negative shift of the I_{DS}-V_{GS} characteristics indicates the p-type CNT channel is more difficult to be turned on because the electrons flow from the $CsPbBr_3$-QD to the p-type CNT and are partially depleted. The sweep swing of V_{GS} is from −5 V to 5 V, and the sweep time is 30 s.

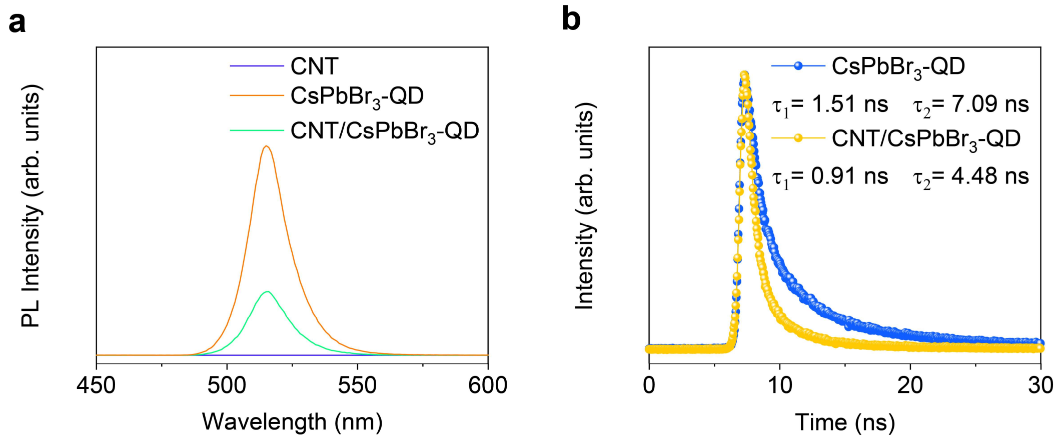

Supplementary Fig. 12 Steady-state and transient photoluminescence (PL) spectra. (a) Steady-state PL spectra of the CNT, $CsPbBr_3$-QD and CNT/$CsPbBr_3$-QD films. Excited by 400 nm photo signal, both the $CsPbBr_3$-QD and CNT/$CsPbBr_3$-QD films have sharp emission peaks centered at 515 nm. However, with the combination of CNTs and $CsPbBr_3$-QDs, the PL intensity becomes weaker, which is due to charge transfer between the $CsPbBr_3$-QD and CNT, indicating that highly effective splitting of excitons has been achieved. (b) Transient PL spectra of the $CsPbBr_3$-QD and CNT/$CsPbBr_3$-QD films. PL decay dynamics are fitted with a double-exponential function to obtain fluorescent lifetime parameters. According to the mean lifetime equation $\tau = \Sigma_i A_i \tau_i^2 / \Sigma_i A_i \tau_i$, where τ_i is the lifetime constant and A_i is the corresponding weight factor, the fast and slow components of the PL lifetime, which are related to the nonradiative and radiative recombination, can be extracted as τ_1 = 1.51 ns and τ_2 = 7.09 ns, and τ_1 = 0.91 ns and τ_2 = 4.48 ns for the $CsPbBr_3$-QD layer and CNT/$CsPbBr_3$-QD film, respectively. The longer lifetime of the $CsPbBr_3$-QDs is consistent with its stronger fluorescence intensity. The fast exciton dissociation at the CNT/$CsPbBr_3$-QD interface contributes to reduce exciton recombination, thereby shortening the lifetime. The

weight factor "A_i" is derived from the lifetime decay fitting, as a mathematical operation, it represents the ratio of related lifetime "τ_i". Considering that the classification of multi-lifetime remains challenging, the average lifetime is usually used to compare the exciton populations, that is, the longer the lifetime, the higher exciton recombination density.

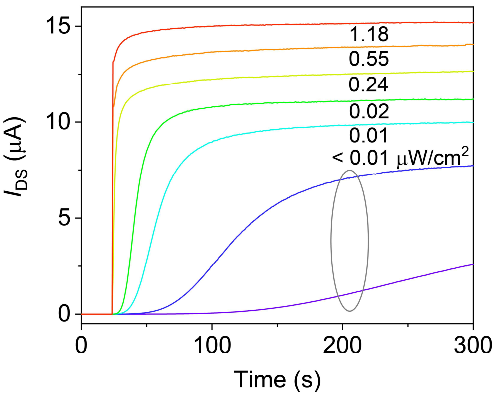

Supplementary Fig. 13 Light-dose-dependent response of the phototransistor at various lighting power densities. The photodetector can response to a weaker light with a lighting power density lower than 0.01 μW/cm^2, which is the limited resolution of our current measurement equipment. The light-dose-dependent response includes time dependence and light intensity dependence. For the time-dependent characteristics, as the light irradiation time is extended, the photocurrent of the phototransistor increases and reaches saturation after a sufficient illumination duration. These phenomena can also be observed in the literature[11, 12]. The time-dependent characteristics of the phototransistor at various lighting power densities during an illumination duration of 275 s, which indicates that the I_{DS} will reach a saturation value as the illumination continues. V_{DS} = 1 V, V_{GS} = 5 V, λ = 405 nm.

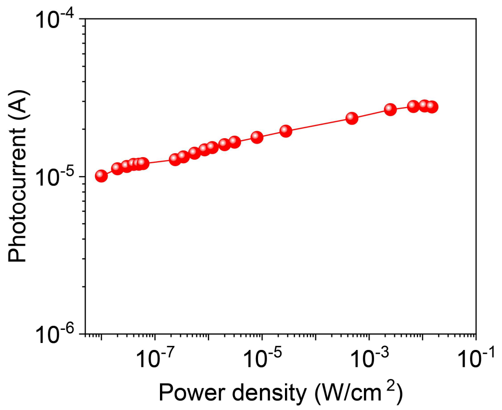

Supplementary Fig. 14 Dependence of the photocurrent (I_{ph}) of the phototransistor on the lighting power density with a 405 nm light illumination. $I_{ph} = I_{light} - I_{dark}$, where I_{light} and I_{dark} are the drain current in the dark and illuminated conditions, respectively.

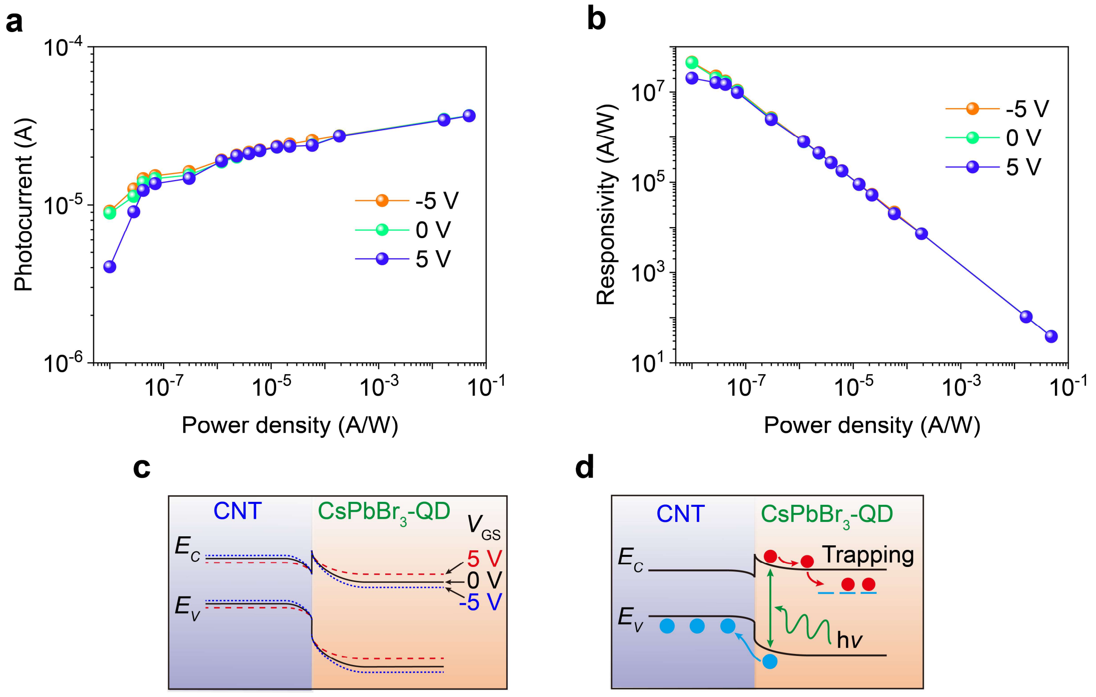

Supplementary Fig. 15 Analysis of optoelectronic characteristics with various gate voltages. (a) Dependence of the photocurrent on *P*. (b) Dependence of responsivity on *P*. It can be found that the photocurrent and responsivity are very similar, especially in the cases of higher lighting power densities, which indicates that the negatively-charged QDs is the dominant factor leading to the increase in current. Therefore, the photocurrent mainly depends on the lighting power density and has no obvious relationship with the gate voltage. (c) Energy band diagram under dark condition at different V_{GS}. A built-in electric field induces a band bending at the CNT/$CsPbBr_3$-QD interface and reaches the Fermi level equilibrium at different V_{GS} (−5, 0 and +5 V) under dark conditions. The built-in electric field is the strongest for V_{GS} = −5 V, because the direction of the electric field of V_{GS} = −5 V is consistent with the built-in electric field, but for V_{GS} = 5 V, the situation is reversed. (d) Energy band diagram under illuminated condition. The electrons are trapped in the QDs resulting in a photogating effect, which

modulates the CNT into ON-state to increase the current. The photocurrent depends on the number of electrons trapped in the QDs, therefore, there is no obvious difference in the energy band diagram under different V_{GS} conditions.

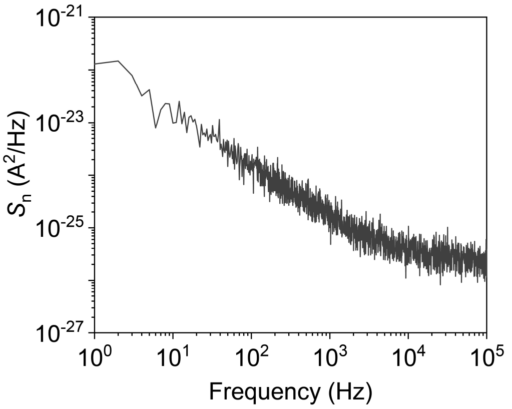

Supplementary Fig. 16 Noise power density (S_n) of the phototransistor. To characterize the actual performance of the CNT/$CsPbBr_3$-QD hybrid, the spectral noise power of the phototransistor has to be recorded. The measured noise in the dark current shows a strong 1/f component. Measured at a frequency of 1 Hz, this leads to a S_n of 1.3×10^{-22} A^2/Hz. V_{DS} = 1 V, V_{GS} = 5 V.

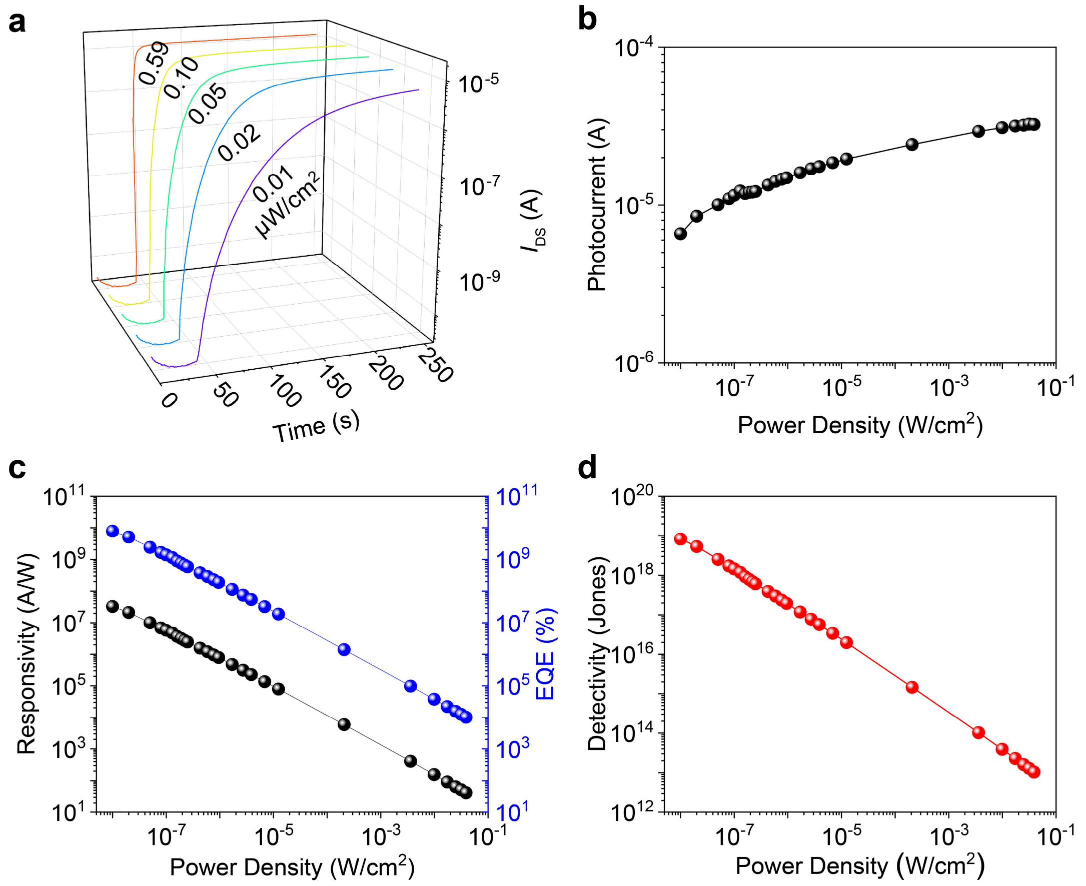

Supplementary Fig. 17 Optoelectronic performance using a light with a wavelength of 516 nm. (a) Light-dose-dependent response of the optoelectronic transistor at various lighting power densities (*P*). (b) Dependence of the photocurrent on *P*. (c) Dependence of the responsivity (*R*) and the external quantum efficiency (EQE) on *P*. $R = 3.3\times10^7$ A/W, EQE = 7.9×10^9 % at $P = 0.01$ μW/cm^2. (d) Dependence of the specific detectivity (D^*) on *P*. $D^* = 1.3\times10^{16}$ Jones at $P = 0.01$ μW/cm^2.

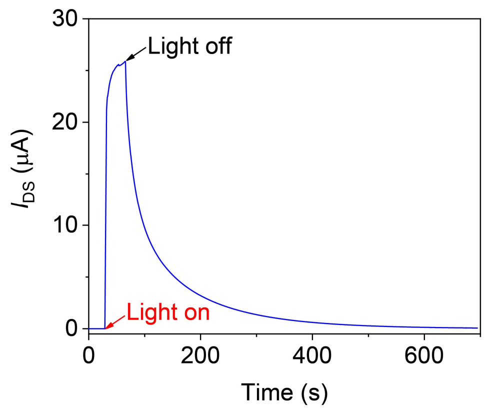

Supplementary Fig. 18 Time-resolved response of the phototransistor. The photocurrent increases when the light is turned on and decays after it is removed. It takes a long time for the device to decay to the original dark state.

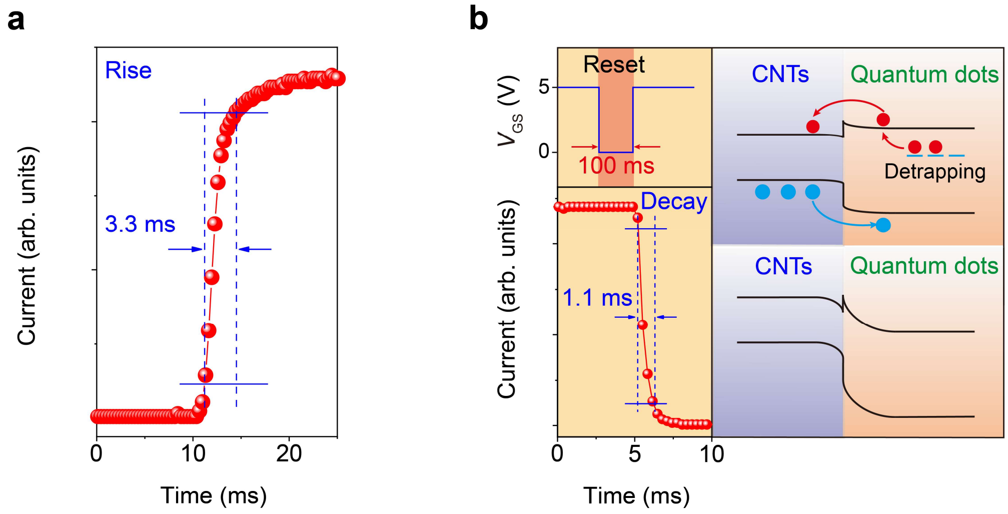

Supplementary Fig. 19 Time-resolved response when the phototransistor is subjected to light and a reset voltage pulse. (a) Temporal response indicates a rise time of 3.3 ms. Rise (decay) time here is defined as the time for the photocurrent to rise (decay) from 10% of the final value to 90% (90 to 10%). $\lambda = 405$ nm, $P = 0.78$ W/cm^2. (b) Decay time of the phototransistor after the laser is turned off and a reset pulse from 5 V to 0 V for 100 ms is used. The decay time is reduced to 1.1 ms. More strictly, the decay time is the period calculated after the completion of the electrical reset pulse. Such operation method is usually used to improve the decay time of phototransistors in the literature[8, 13, 14]. Energy bands of CNT/$CsPbBr_3$-QD interface are shown by applying a reset pulse which accelerates the detrapping process. This pulse generates an electric field that induces many positive carriers in the CNT film, which attracts trapped electrons escaping to the CNT layer, resulting in recombination, followed by a rapid decay of the photocurrent (top panel). Equilibrium of the energy bands of the CNT/$CsPbBr_3$-QD interface after the detrapping process is finished is shown in the bottom panel.

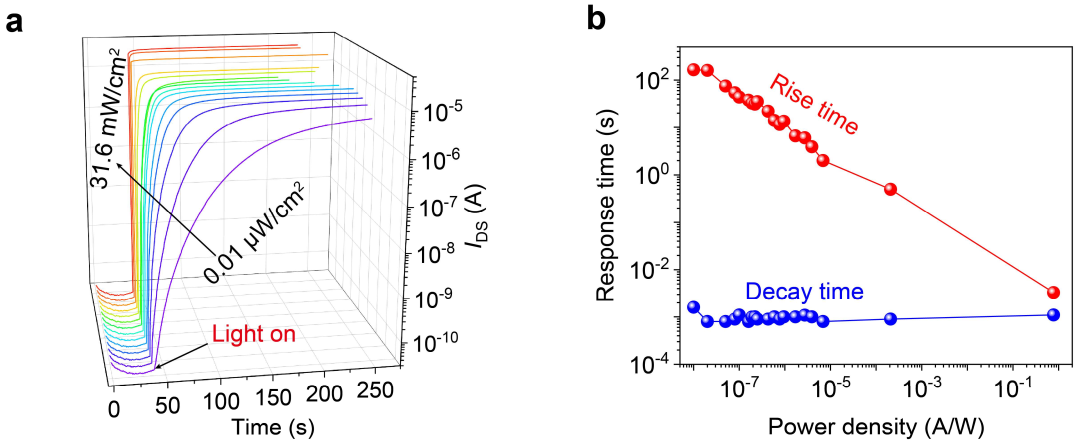

Supplementary Fig. 20 Dependence of the response time on the lighting power density. (a) Light-dose-dependent response of the optoelectronic transistor at various lighting power densities (P). The less time is required to reach photocurrent saturation with higher lighting power density. (b) Dependence of the rise and decay time on P. The rise time can decrease to 3.3 ms at 0.78 W/cm^2, and the decay time is about 1 ms which is not associated with lighting power density. V_{DS} = 1 V, V_{GS} = 5 V, λ = 516 nm.

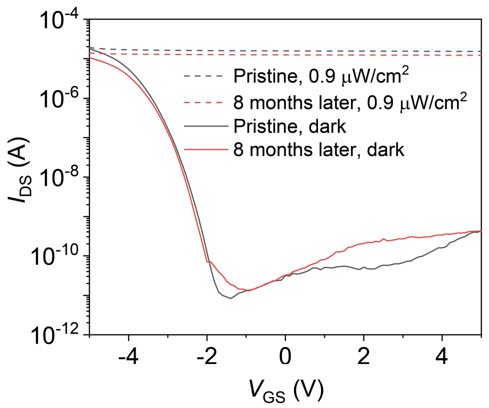

Supplementary Fig. 21 Stability of the electrical and optoelectronic performance of phototransistor at room temperature. The transfer curves under dark and illuminated conditions (0.9 μW/cm^2) were recorded for 8 months. Based on the excellent stability of CNTs and $CsPbBr_3$-QDs, the maintained optoelectronic characteristics indicate the long-term stability of photocurrent. V_{DS} = 1 V, λ= 405 nm.

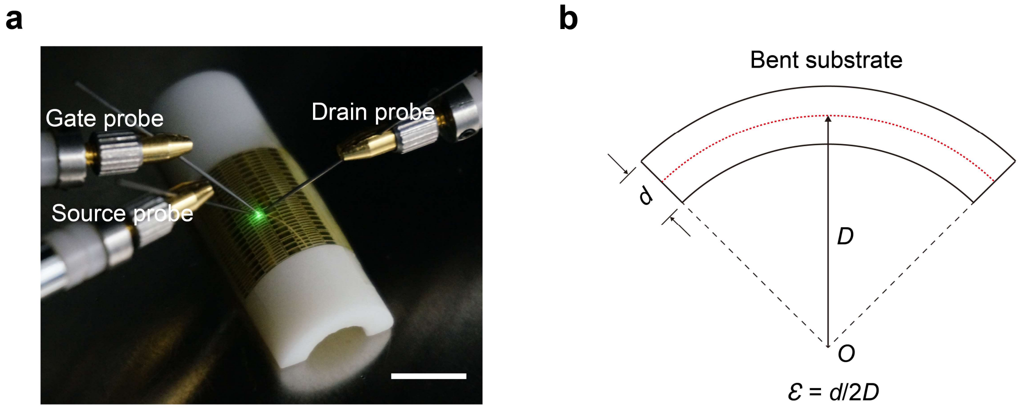

Supplementary Fig. 22 Experimental setup and schematic of a bent substrate for the characterization of phototransistor flexibility. (a) Photograph of a phototransistor on a bent substrate (scale bar, 1 cm). (b) Schematic of the bent substrate. The radius of curvature (*D*) is measured from the point of origin (*O*) to the neutral axis of the substrate. The bending strain ε is $d/2D$, where *d* is the thickness of the device.

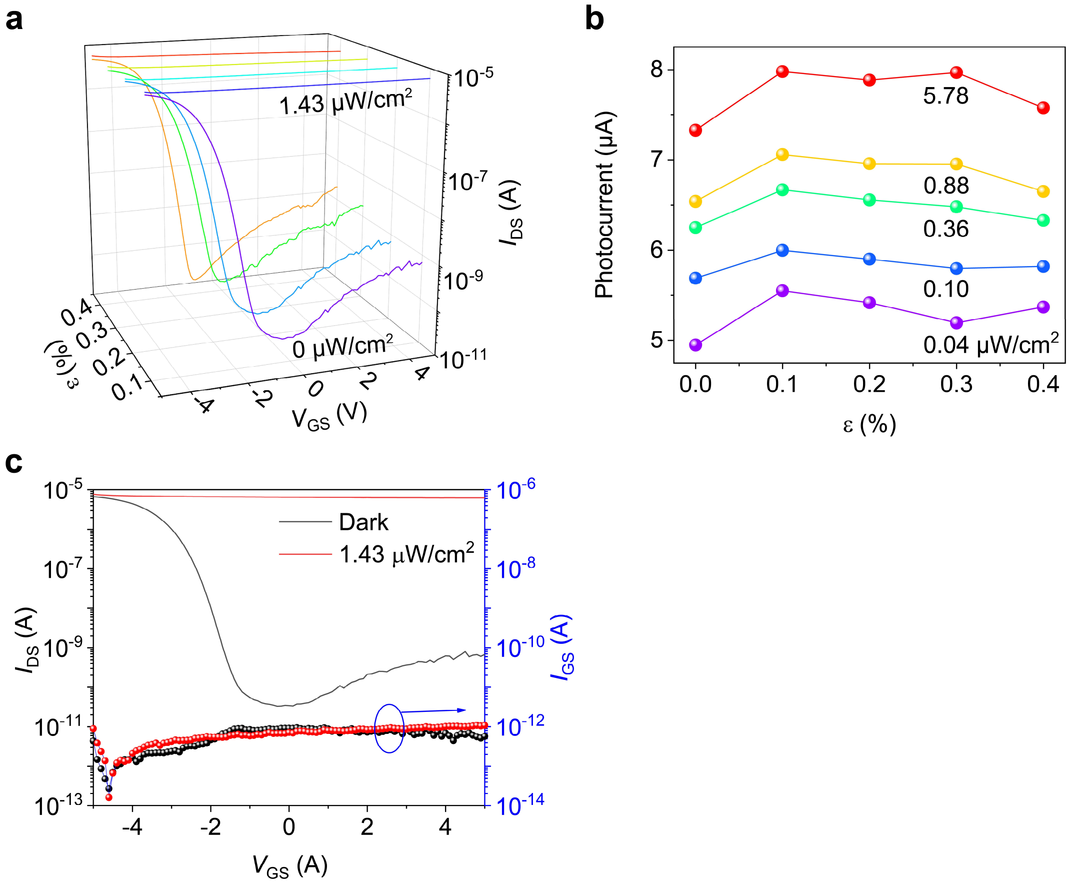

Supplementary Fig. 23 Bending measurements of the flexible phototransistor. (a) I_{DS}-V_{GS} of the flexible device given different bending strains in dark and lighted (516 nm, 1.43 μW/cm^2) conditions. (b) Stability test of the photocurrent at different bending strains and lighting power densities. (c) I_{DS}-V_{GS} and I_{GS}-V_{GS} characteristics of the flexible device given a fixed bending strain (ε = 0.4%) under dark and illuminated conditions (P = 1.43 μW/cm^2), and the sub-picoampere leakage current demonstrates the robustness of the device. V_{DS} = 1 V.

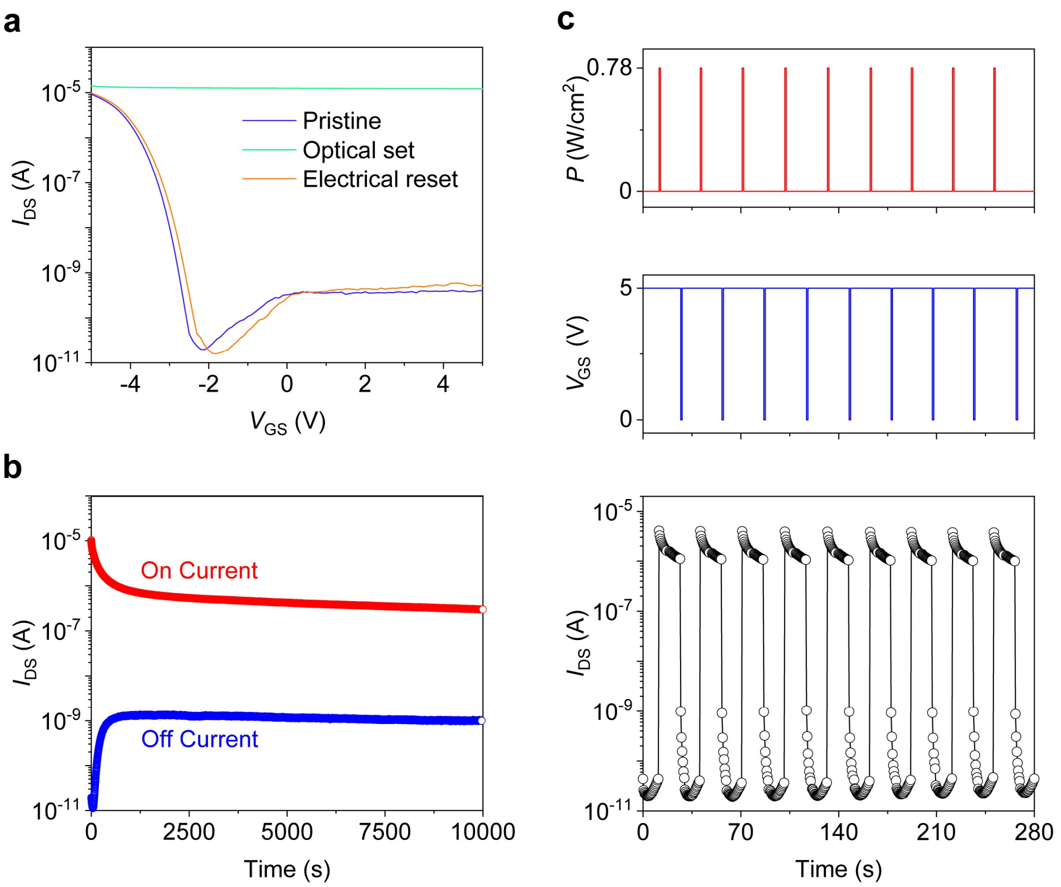

Supplementary Fig. 24 Memory performance of the phototransistor. (a) Transfer characteristics of the device: pristine in the dark, after being exposed to 516 nm illumination at 0.208 mW/cm^2 for 20 s to conduct an optical set operation, and after using an electrical pulse of 0 V for 100 ms as electrical reset operation. (b) Retention test of pulse switching. The set process was operated by a light (516 nm) pulse with a lighting power density of 0.78 W/cm^2 and pulse width of 10 s. An electrical pulse (0 V, 100 ms) is used for the reset process. V_{DS} = 1 V, V_{GS} = 5 V. (c) Pulse-switching characteristics. An optical pulse (516 nm) with a lighting power density of 0.78 W/cm^2 and a pulse width of 500 ms was used for the set process, and the reset process was initiated by an electrical pulse (from 5 V to 0 V, 100 ms).

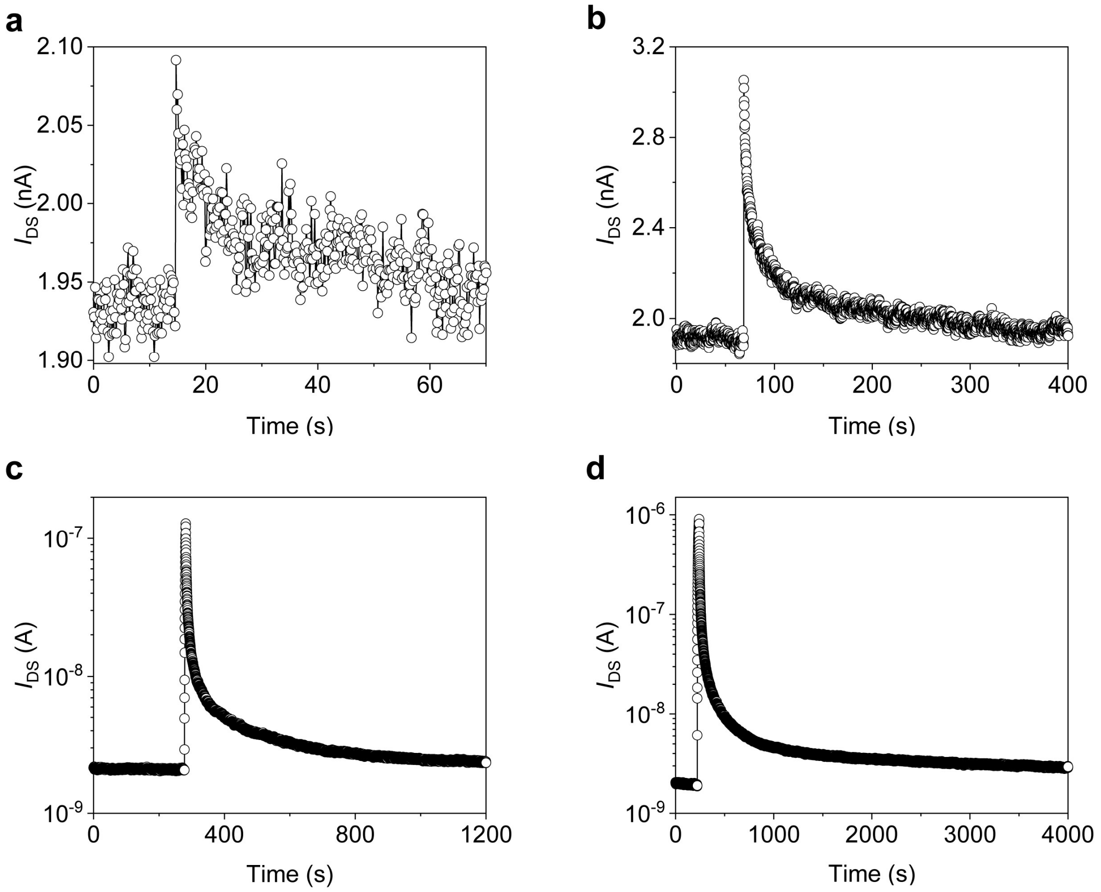

Supplementary Fig. 25 The transition from short-term plasticity (STP) to long-term plasticity (LTP). (a) STP with a light pulse width of 10 ms (516 nm, 6.8 μW/cm^2). V_{DS} = 1 V, V_{GS} = 5 V. (b) LTP with a pulse width of 100 ms. (c) LTP with a pulse width of 4000 ms. (d) LTP with a pulse width of 10000 ms. According to the light-dose-dependent response characteristics, the phototransistor is switched to a more conductive state if a longer pulse width with the same *P* is given, giving rise to an enhanced synaptic strength which includes a large spike current and a long relaxation time. The relaxation time increases from tens of seconds with a pulse width of 10 ms to thousands of seconds with a pulse width of 10000 ms.

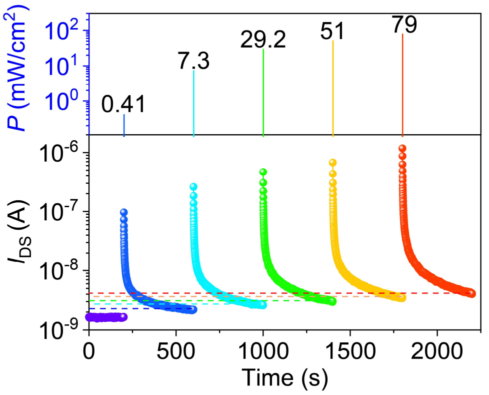

Supplementary Fig. 26 Light-intensity-dependent synaptic plasticity with a pulse width of 20 ms. This demonstrates the consecutive changes of current with decay properties under stimulation of a sequence of precise optical pulses with various intensities. After 400 s decay, increases in current values of 0.56, 0.98, 1.37, 1.87, 2.42 nA are observed compared with the original current (1.65 nA) when the lighting power density increases from 0.41, 7.3, 29.2, 51 to 79 mW/cm^2. Thus, volatile-to-nonvolatile memory is transformed during the cumulative modulation with optical pulses, which stimulates the transformation of the synaptic plasticity from STP to LTP.

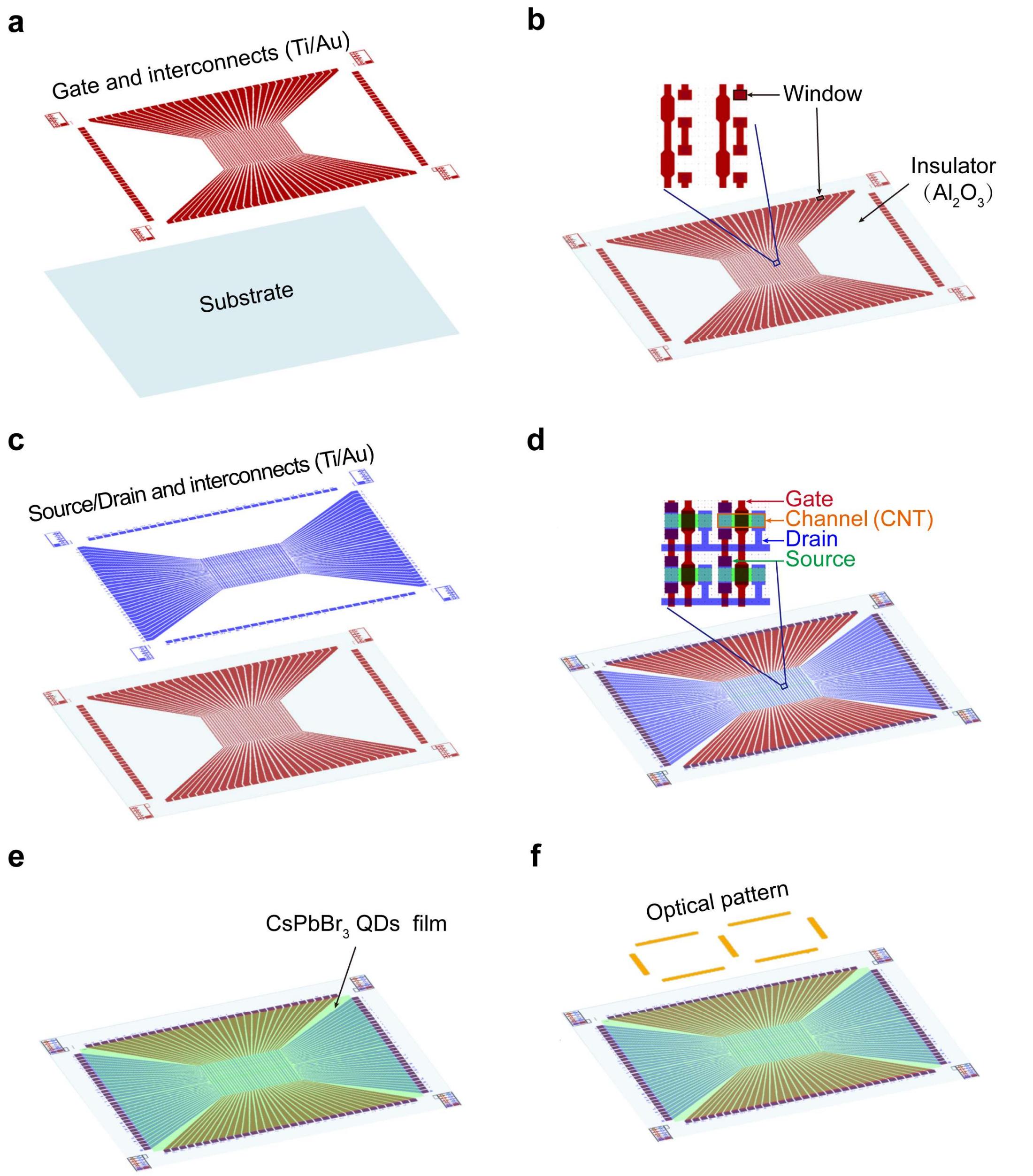

Supplementary Fig. 27 Fabrication of the optoelectronic sensor array. (a) Fabrication of the gate electrodes and interconnects (Ti/Au: 5/50 nm) deposited on a PEN substrate. (b) Insulator deposition and contact window formation. An 80-nm-thick insulating Al_2O_3 layer was deposited on the substrate by ALD, followed by the opening of a window by a wet etching process using phosphoric acid at 70 °C for 6 min. (c) Fabrication of the source/drain electrodes and interconnects (Ti/Au: 5/50 nm). (d) Deposition and patterning of the solution-processed CNT channel. (e) Spin-coating the

$CsPbBr_3$-QD film. (f) Shadow mask. A metal layer was deposited onto transparent glass to form an “8” pattern for light sensing.

a b c

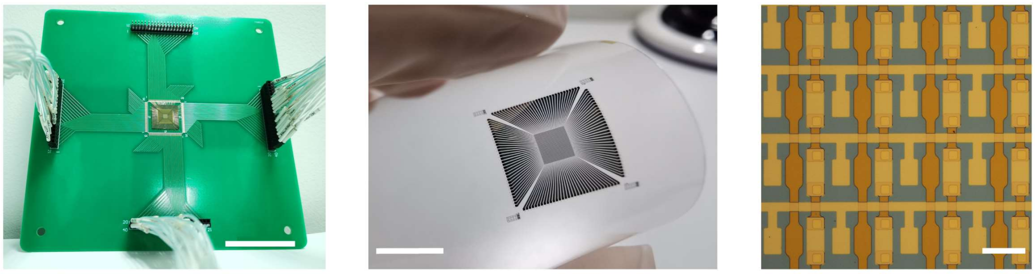

Supplementary Fig. 28 Optical photographs of the optoelectronic sensor array. (a) Photograph of a sensor array mounted on a PCB (scale bar, 40 mm). (b) Photograph of the flexible sensor array fabricated (scale bar, 5 mm). (c) Magnified image of part of the core of the chip (scale bar, 50 μm).

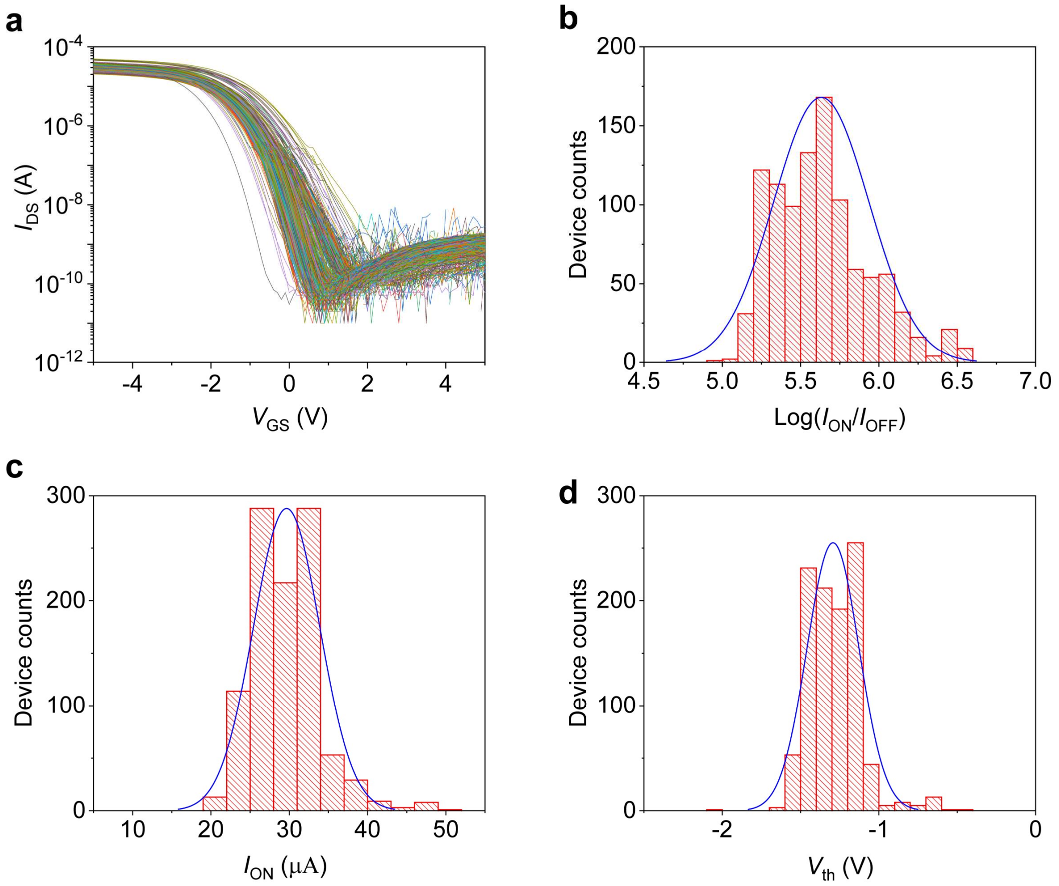

Supplementary Fig. 29 Statistical analysis of the devices in the array. (a) The typical transfer characteristics of 1024 pixels were measured under dark conditions and these pixels show a uniform electrical performance at V_{DS} = 1 V. (b) The statistical distribution of on/off ratio is fitted with a logarithmic normal curve with a variance of 5.6%. (c) The on current (I_{ON}) statistical distribution with a variance of 13.9%. (d) Threshold voltage (V_{th}) statistical distribution with a variance of 14.7%.

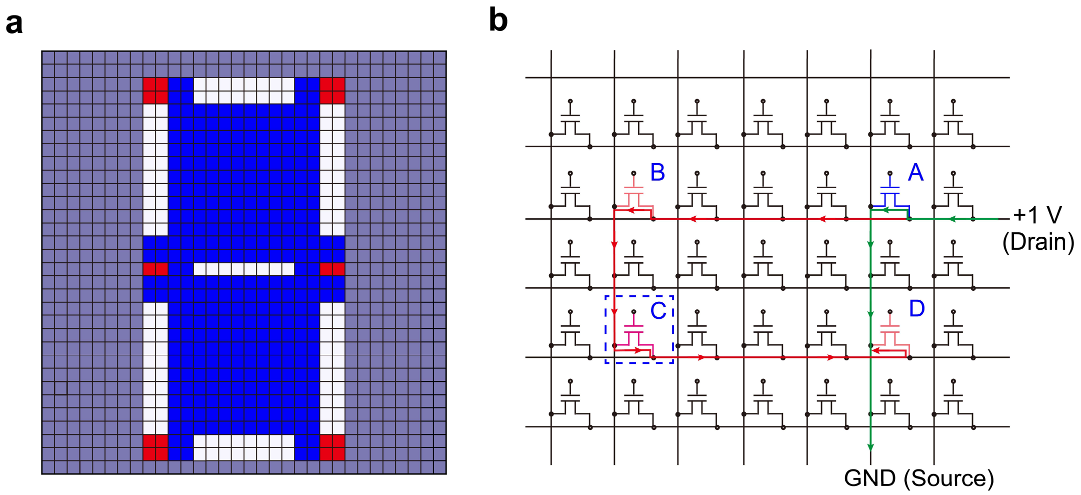

Supplementary Fig. 30 Designed principle avoiding crosstalk issue. The sensor array with one-transistor configuration may lead to crosstalk issue between neighboring sensor units if the neighboring devices remain the ON state. In order to measure number "8" pattern, we designed a special mask shown in Supplementary Fig. 30a (the devices in the white region are ON state). If the devices in red region remain OFF state (dark condition), the blue area can avoid crosstalk issues. For an instance, the device A is measuring phototransistor which remains OFF state, and the device B and D are neighboring phototransistors which remain the ON state (Supplementary Fig. 30b). The green line represents the actual current value of device A, and the red line represents the crosstalk current flowing through devices B, C and D. In our design, we designed a special mask to keep device C in OFF state to avoid crosstalk issues. In order to achieve more complex images in future, such as face images with finer and more complex features, and super-resolution for face images is more challenging without the selector transistor or diode.

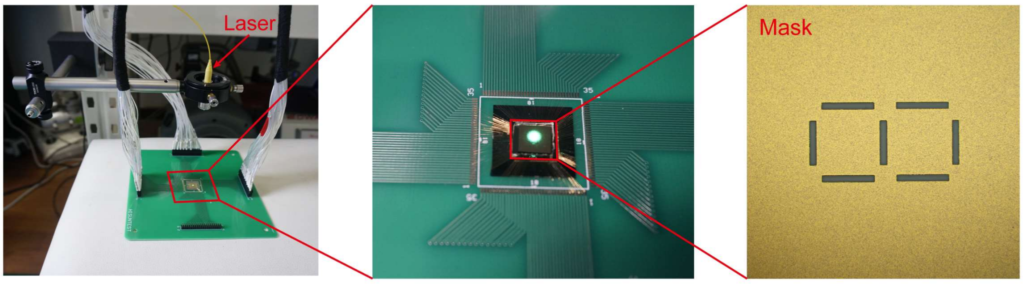

Supplementary Fig. 31 Photographs of the optical setup. The pattern was formed from a shadow mask with a transparent number "8" during optical pulse illumination.

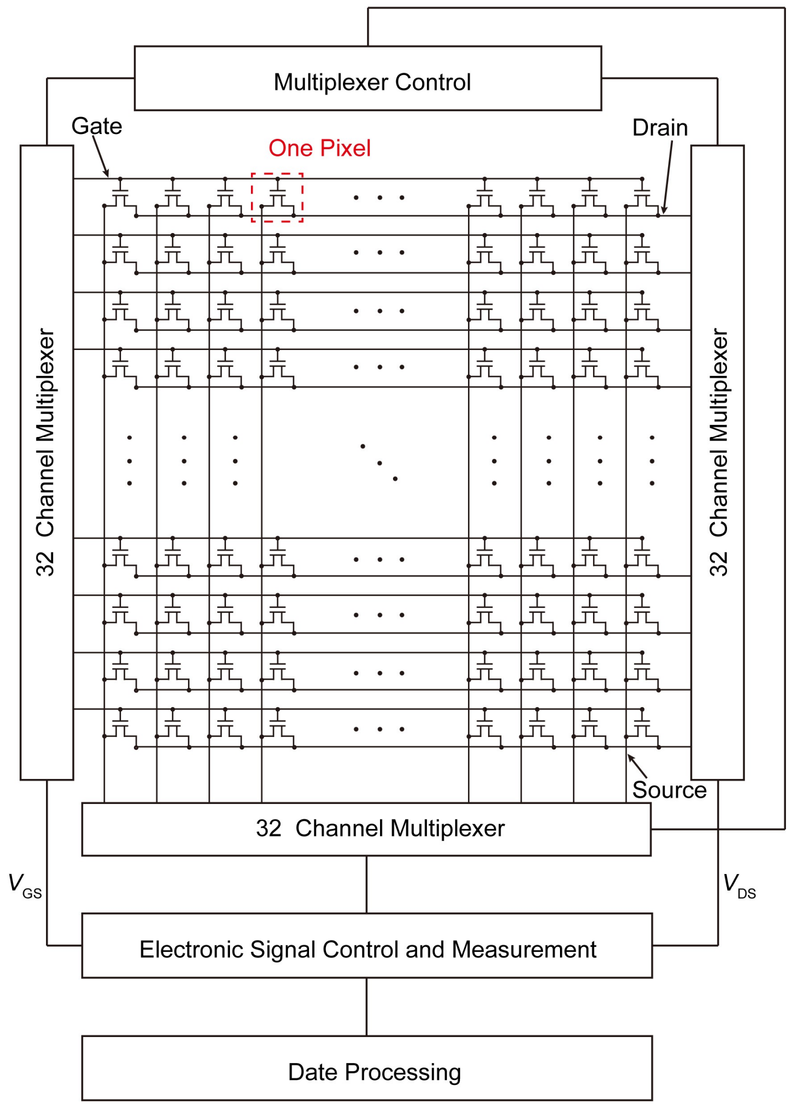

Supplementary Fig. 32 Principle of the measurement system for the optoelectronic sensor array. The measurement process is introduced as follows. The special mask was put on the sensor array and the laser is turned on (Supplementary Fig. 30). Next, all 1024 devices were switched off by applied $V_{GS} = 5$ V to the 32 gate channel multiplexers. Finally, the I_{DS} of each pixel in the sensor array were measured by sequentially connecting or disconnecting the circuit through the multiplexer. The time required to achieve a measured pattern depends on the time to measure I_{DS} of one pixel. For example, in order to show an ordinary pattern, it takes less than 1 s for one pixel, and it

takes about 1000 s in total.

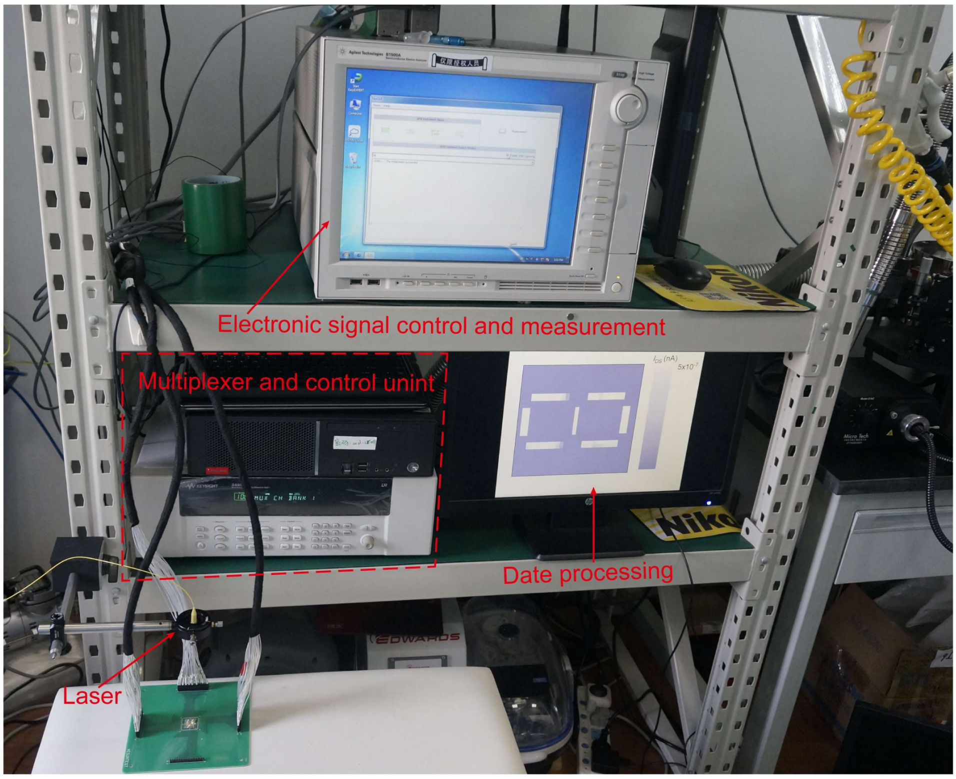

Supplementary Fig. 33 Photograph of the measurement system for the optoelectronic sensor array. The system contains four parts: (i) laser, (ii) multiplexer and control unit, (iii) electrical signal control and measurement, and (iv) data processing.

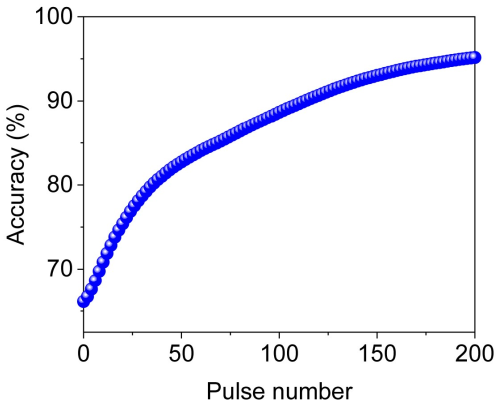

Supplementary Fig. 34 Training accuracy as a function of number of training pulses. Training accuracy is defined as: $\text{Accuracy} = \left[1 - \sqrt{\sum_{i=1}^{m} \frac{(I_{\text{target}}(i) - I_{\text{trained}}(i)}{m}}\right] \times 100\%$, where $I_{\text{target}}(i)$ and $I_{\text{trained}}(i)$ are the target and trained current, respectively, at the *i*th synapse in an array with *m* synapses, and the current values are normalized to the total plasticity from 0 to 1. The root mean square in the equation calculates the average discrepancy between the ideal number "8" pattern and the trained weight map. The calculated accuracy as a function of the training number of pulses is illustrated. This optical training scheme achieved 95% training accuracy for 200 pulses.

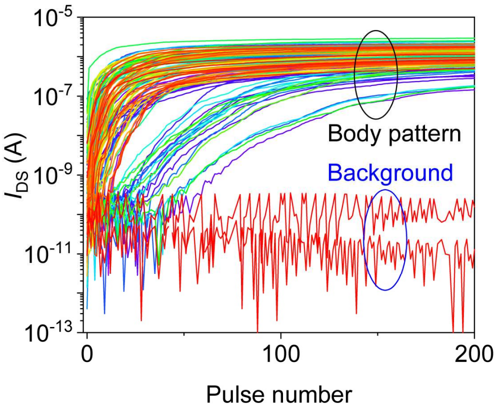

Supplementary Fig. 35 Long-term potentiation of the sensor array with 200 optical pulses (pulse width, 250ms; pulse interval, 250 ms). λ = 405 nm, P = 1 μW/cm^2, V_{GS} = 5 V, V_{DS} = 0.5 V. For body pattern pixels ("8" pattern), which contains 120 devices, the I_{DS} increases steadily with the number of training pulses. For background pixels, I_{DS} remains at the dark current level.

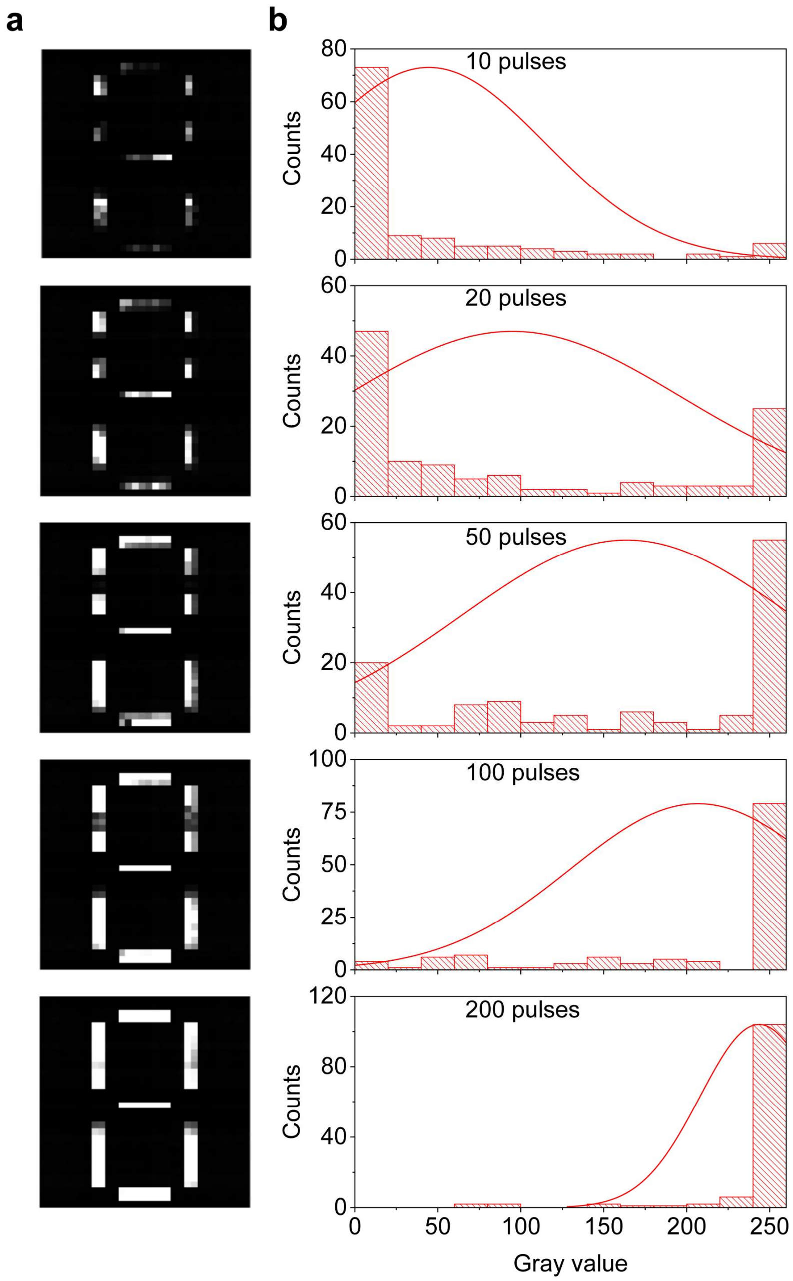

Supplementary Fig. 36 Distribution of the gray value of body pixels after training for 10, 20, 50, 100 and 200 pulses. (a) The current of training weight maps of the number “8” pattern for different numbers of training pulses in Fig. 3e is transferred to a gray value. Current values greater than or equal to 500 nA are converted to a gray value of 255. (b) The distribution of gray values corresponding to Supplementary Fig. 36a.

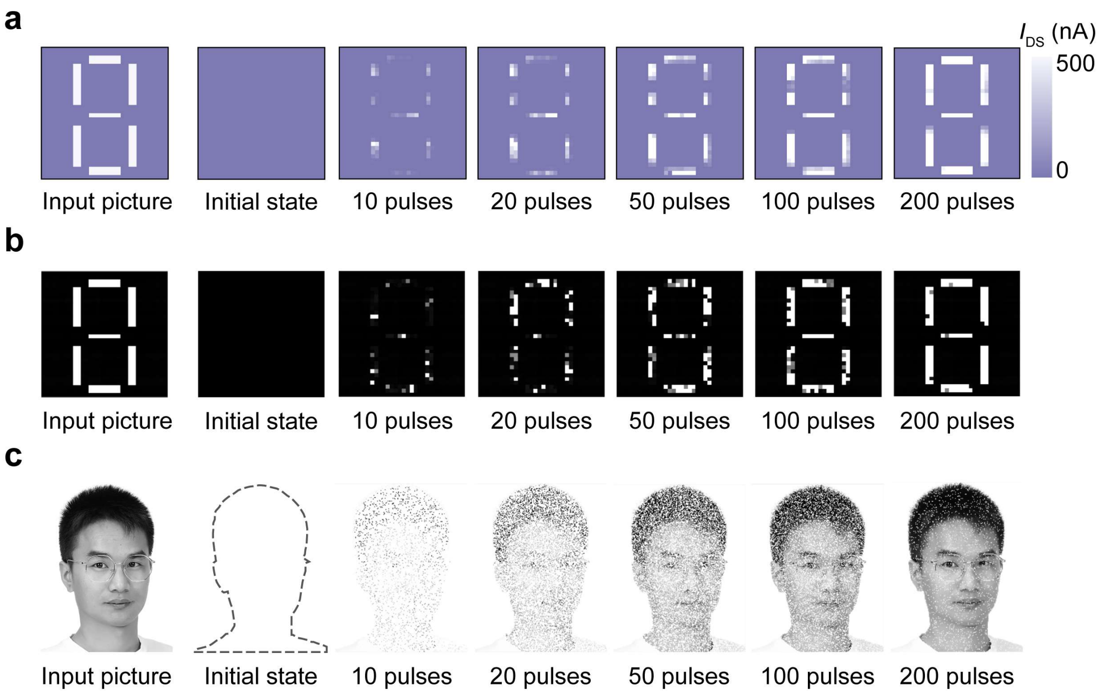

Supplementary Fig. 37 Evaluation of images during the training process for the initial and different learning states based on experimental results and simulation. (a) The experimental results of a 32 × 32 optoelectronic sensor array. (b) Simulation results of an "8" pattern in the initial state and after training. We obtain similar results to the experimental ones. (c) Simulation results of a face in the initial state and after training.

References

1. Ding, J. et al. Enrichment of large-diameter semiconducting SWCNTs by polyfluorene extraction for high network density thin film transistors. *Nanoscale* **6**, 2328-2339 (2014).
2. Gu, J. et al. Solution-processable high-purity semiconducting SWCNTs for large-area fabrication of high-performance thin-film transistors. *Small* **12**, 4993-4999 (2016).
3. Liu, L. et al. Aligned, high-density semiconducting carbon nanotube arrays for high-performance electronics. *Science* **368**, 850-856 (2020).
4. Weisman, R. B. & Bachilo, S. M. Dependence of optical transition energies on structure for single-walled carbon nanotubes in aqueous suspension: an empirical Kataura plot. *Nano Lett.* **3**, 1235-1238 (2003).
5. Javey, A. et al. Ballistic carbon nanotube field-effect transistors. *Nature* **424**, 654-657 (2003).
6. Suzuki, S., Bower, C., Watanabe, Y. & Zhou, O. Work functions and valence band states of pristine and Cs-intercalated single-walled carbon nanotube bundles. *Appl. Phys. Lett.* **76**, 4007-4009 (2000).
7. Xiao, M. et al. Surface ligands stabilized lead halide perovskite quantum dot photocatalyst for visible light-driven hydrogen generation. *Adv. Funct. Mater.* **29**, 1905683 (2019).
8. Konstantatos, G. et al. Hybrid graphene-quantum dot phototransistors with ultrahigh gain. *Nat. Nanotechnol.* **7**, 363-368 (2012).

9. Sun, Z. et al.Infrared photodetectors based on CVD-grown graphene and PbS quantum dots with ultrahigh responsivity. *Adv. Mater.* **24**, 1202220 (2012).

10. Kufer, D. & Konstantatos, G. Photo-FETs: phototransistors enabled by 2D and 0D nanomaterials. *ACS Photonics* **3**, 2197–2210 (2016).

11. Zhou, F. et al. Optoelectronic resistive random access memory for neuromorphic vision sensors. *Nat. Nanotechnol.* **14**, 776-782 (2019).

12. Liu, X. et al. All-printable band-edge modulated ZnO nanowire photodetectors with ultra-high detectivity. *Nat. Commun.* **5**, 4007 (2014).

13. Jeon, S. et al. Gated three-terminal device architecture to eliminate persistent photoconductivity in oxide semiconductor photosensor arrays. *Nat. Mater.* **11**, 301-305 (2012).

14. Lopez-Sanchez, O. et al. Ultrasensitive photodetectors based on monolayer MoS_2. *Nat. Nanotechnol.* **8**, 497-501 (2013).
